# Rituximab therapy in pulmonary alveolar proteinosis improves alveolar macrophage lipid homeostasis

**DOI:** 10.1186/1465-9921-13-46

**Published:** 2012-06-14

**Authors:** Anagha Malur, Mani S Kavuru, Irene Marshall, Barbara P Barna, Isham Huizar, Reema Karnekar, Mary Jane Thomassen

**Affiliations:** 1 Program in Lung Cell Biology and Translational Research, Division of Pulmonary, Critical Care and Sleep Medicine, East Carolina University, Greenville, NC, USA; 2 Division of Pulmonary, Critical Care and Sleep Medicine, East Carolina University, Brody School of Medicine, 3E-149 Brody Medical Sciences Building, Greenville, NC, 27834, USA; 3 Current address: Division of Pulmonary & Critical Care Medicine, Thomas Jefferson University & Hospital, 834 Walnut St., Suite 650, Philadelphia, PA, 19107, USA

**Keywords:** PAP, Rituximab, Alveolar macrophages, Surfactant, PPARγ, ABCG1, LPLA2

## Abstract

**Rationale:**

Pulmonary Alveolar Proteinosis (PAP) patients exhibit an acquired deficiency of biologically active granulocyte-macrophage colony stimulating factor (GM-CSF) attributable to GM-CSF specific autoantibodies. PAP alveolar macrophages are foamy, lipid-filled cells with impaired surfactant clearance and markedly reduced expression of the transcription factor peroxisome proliferator-activated receptor gamma (PPARγ) and the PPARγ-regulated ATP binding cassette (ABC) lipid transporter, ABCG1. An open label proof of concept Phase II clinical trial was conducted in PAP patients using rituximab, a chimeric murine-human monoclonal antibody directed against B lymphocyte specific antigen CD20. Rituximab treatment decreased anti-GM-CSF antibody levels in bronchoalveolar lavage (BAL) fluid, and 7/9 patients completing the trial demonstrated clinical improvement as measured by arterial blood oxygenation.

**Objectives:**

This study sought to determine whether rituximab therapy would restore lipid metabolism in PAP alveolar macrophages.

**Methods:**

BAL samples were collected from patients pre- and 6-months post-rituximab infusion for evaluation of mRNA and lipid changes.

**Results:**

Mean PPARγ and ABCG1 mRNA expression increased 2.8 and 5.3-fold respectively (p ≤ 0.05) after treatment. Lysosomal phospholipase A2 (LPLA2) (a key enzyme in surfactant degradation) mRNA expression was severely deficient in PAP patients pre-treatment but increased 2.8-fold post-treatment. In supplemental animal studies, LPLA2 deficiency was verified in GM-CSF KO mice but was not present in macrophage-specific PPARγ KO mice compared to wild-type controls. Oil Red O intensity of PAP alveolar macrophages decreased after treatment, indicating reduced intracellular lipid while extracellular free cholesterol increased in BAL fluid. Furthermore, total protein and Surfactant protein A were significantly decreased in the BAL fluid post therapy.

**Conclusions:**

Reduction in GM-CSF autoantibodies by rituximab therapy improves alveolar macrophage lipid metabolism by increasing lipid transport and surfactant catabolism. Mechanisms may involve GM-CSF stimulation of alveolar macrophage ABCG1 and LPLA2 activities by distinct pathways.

## Introduction

Surfactant catabolism is impaired in alveolar macrophages from patients with pulmonary alveolar proteinosis (PAP) and GM-CSF knock out (KO) mice. In the GM-CSF KO mouse, the PAP like syndrome is reversible by exogenous GM-CSF or local over-expression of GM-CSF. Studies from GM-CSF KO mice initially suggested that PAP might be due to idiopathic defects in GM-CSF receptors or production [1]. In adults with PAP, however, no mutations in GM-CSF receptor or surfactant coding sequences have been described [2]. Moreover, studies from our lab and others reported that both monocytes and alveolar macrophages from adult PAP patients are able to produce and respond to GM-CSF [3,4]. Evidence for adult PAP as an autoimmune disease was first presented by Kitamura et al., who noted that circulating anti-GM-CSF autoantibodies neutralized GM-CSF biological activity, and thus resulted in a virtual GM-CSF deficiency [5,6]. Subsequent studies in idiopathic adult PAP patients confirmed the existence of anti-GM-CSF antibodies and demonstrated that autoantibody levels were clinically useful for diagnosis [7-10].

Alveolar macrophages from both PAP patients and GM-CSF KO mice display a striking deficiency in PPARγ and in the lipid transporter ABCG1 [11,12]. GM-CSF treatment increased ABCG1 expression in macrophages *in vitro* and in alveolar macrophages of PAP patients *in vivo*. Overexpression of PPARγ by lentivirus-PPARγ transduction of primary human alveolar macrophages or activation by rosiglitazone also increased ABCG1 expression [12]. In GM-CSF KO mice, in *vivo* treatment with lentivirus-PPARγ increased both PPARγ and ABCGI expression while reducing lipid accumulation in the lung. More recently, we observed improved lung function and reduced lipid accumulation in GM-CSF KO mice treated *in vivo* with lentivirus-ABCG1 [13]. Collectively, these studies suggest that surfactant accumulation in PAP alveolar macrophages stems from GM-CSF deficiency leading to PPARγ deficiency and subsequent reduction of ABCG1 expression.

Lung surfactant catabolism is also known to be dependent upon lysosomal phospholipase A2 (LPLA2) activity, an enzyme selectively expressed in alveolar macrophages but not other tissue macrophages or circulating monocytes [14]. LPLA2 activity is deficient in GM-CSF KO mice but is restored by transgenic expression of GM-CSF [14]. Interestingly, *in vitro* studies provide no evidence of LPLA2 stimulation by PPARγ although induction occurs through the PPARγ heterodimer, retinoid X receptor (RXR) via stimulation by all-trans-retinoic acid [15].

Rituximab, a chimeric murine-human monoclonal antibody directed against CD20, a B lymphocyte-specific membrane antigen, has been shown to deplete human B cells *in vivo*[16]. Rituximab was approved by the Food and Drug Administration in 1997 for treatment of CD20(+) B cell lymphoma and has since been in use for B cell malignancies. Subsequently, rituximab therapy was applied to treatment of autoimmune disease, and results have shown clinical benefit in systemic lupus erythematosus [17], rheumatoid arthritis [18], and Wegener’s granulomatosis [19] among others.

Based upon data indicating autoantibody involvement in PAP, we recently carried out the first prospective, open label, proof of concept trial of rituximab in ten patients with PAP. Results suggest that rituximab may be an effective primary therapy in this autoimmune disease [20]. The most striking clinical finding from our PAP study of rituximab was the significant improvement of oxygenation, the primary endpoint in 7/9 patients. Improvements were also noted in total lung capacity, HRCT scans and transitional dyspnea index. Importantly, neither total serum anti-GM-CSF nor serum GM-CSF neutralizing capacity were reduced following rituximab therapy. Reduction of anti-GM-CSF levels in BAL fluid from the lung, however, did correlate with improvement in PaO_2_ and HRCT scans.

Mechanisms responsible for rituximab-mediated improvement in PAP disease activity are unclear. Our previous studies of alveolar macrophages from untreated PAP patients demonstrated dramatically reduced PPARγ and ABCG1 expression that was reversible by either *in vivo* or *in vitro* GM-CSF treatment [11,12]. In GM-CSF KO mice, *in vivo* administration of lentivirus constructs containing either PPARγ or ABCG1 reduced alveolar macrophage lipid accumulation and upregulated PPARγ and ABCG1 [13,21]. Based on such observations, we hypothesized that the clinical improvement in rituximab-treated PAP patients might be due to restoration of alveolar macrophage lipid homeostasis associated with reduced GM-CSF autoantibody in the pulmonary compartment. In this paper, we investigated this hypothesis by utilizing BAL samples from the original cohort of PAP patients treated with rituximab.

## Methods

### Study design

This study was a prospective, open-label, proof-of-concept clinical trial of rituximab therapy in a group of 10 adult patients presenting with moderately symptomatic, idiopathic PAP as described in detail [20]. The study was approved by the Institutional Review Board at East Carolina University and informed consent was obtained from all patients. The trial was registered at clinicaltrials.gov (NCT00552461).

### Cell collection

Bronchoalveolar lavage (BAL) was carried out prior to and 6 months after therapy as described [3,20,22]. Cytospins were stained with Oil Red O to detect intracellular neutral lipids and counterstained with Gill’s hematoxylin. Oil Red O intensity was quantified using a modified scoring system previously described [21,23]: +++ = strongly positive; ++ = positive; and + = weakly positive.

### Mice

Animal studies were conducted in conformity with Public Health Service (PHS) Policy on humane care and use of laboratory animals and were approved by the institutional animal care committee. The GM-CSF KO mice were generated by Dr. Glenn Dranoff and have been previously described [24]. Macrophage-specific PPARγ KO mice have been previously described [25]. Animals studied were age (8–12 week old) and gender-matched to wild type C57Bl/6 controls obtained from Jackson Laboratory (Bar Harbor, ME). BAL cells were obtained as described earlier [25]. For all experiments 5–7 mice per group were used.

### RNA purification and analysis

Total cellular RNA was extracted by Lipid RNeasy protocol (Qiagen, Valencia, CA). Expression of mRNA was determined by real time RT-PCR using the ABI Prism 7300 Detection System (TaqMan; Applied Biosystems, Foster City, CA.) according to the manufacturer’s instructions. RNA specimens were analyzed in duplicate using primer/probe sets for human PPARγ, ABCA1, ABCG1, LPLA2 and the housekeeping gene, glyceraldehyde 3 phosphate dehydrogenase (GAPDH) (ABI) or murine LPLA2 and GAPDH (ABI) as previously described [12]. Threshold cycle (CT) values for genes of interest were normalized to GAPDH and used to calculate the relative quantity of mRNA expression. Data were expressed as a fold change in mRNA expression relative to control values [26].

### Cholesterol analysis

Cholesterol was measured in BAL fluid using the Abcam (Cambridge, MA) Cholesterol Assay according to the manufacturer’s instructions. Cholesterol content was expressed as μg/μl of cholesterol.

### Surfactant protein-A (SP-A) ELISA

SP-A was measured in BAL fluid with an ELISA kit (Biovendor, Candler, NC) according to manufacturer’s instructions. Total protein in BAL fluid was measured by BCA protein assay (Pierce, Rockford, MA).

### Statistical analysis

Parametric data are presented as means (±SEM) and nonparametric data are presented as medians and ranges. Statistical comparisons of parametric data were made with Student’s *t* test. Nonparametric data were compared with Wilcoxon test. P ≤ 0.05 was considered significant.

## Results

### PPARγ and ABC lipid transporter expression levels are elevated in alveolar macrophages from patients with PAP

**Figure 1:**
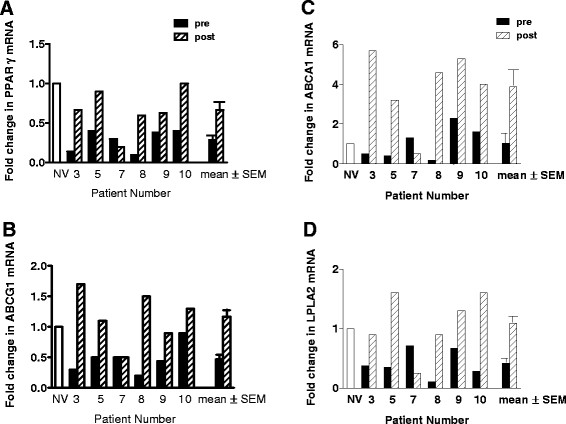
**Gene expression of lipid regulators is increased post-rituximab therapy.** Alveolar macrophage gene expression was measured by RT-PCR in PAP patients before and 6 months post rituximab therapy. Increases are shown in: **(A)** PPARγ; **(B)** ABCG1; **(C)** ABCA1; and **(D)** LPLA2 (n = 6).

Compared to pre-treatment levels, rituximab increased PAP alveolar macrophage PPARγ and ABCG1 mRNA expression by a mean of 2.8 and 5.3-fold respectively (p ≤ 0.05, n = 6) (Figures 1A and B). Although, no significant differences in mRNA expression levels of ABCA1 between healthy controls and PAP baseline alveolar macrophages were found, levels became elevated after rituximab, with a mean increase of 3.2 fold (p < 0.05, n = 6) compared to pre-treatment (Figure1C).

### LPLA2 expression is deficient pre-treatment and elevated by rituximab therapy

Examination of LPLA2, also indicated restorative effects of rituximab. Mean LPLA2 levels were significantly reduced compared to healthy controls in PAP patients pre-treatment (p < 0.05, n = 6). Compared to pre-treatment, however, LPLA2 mRNA expression significantly increased post-rituximab by a mean of 2.8-fold (p < 0.05) (Figure1D).

### LPLA2 expression is not deficient in macrophage-specific PPARγ KO mice

**Figure 2:**
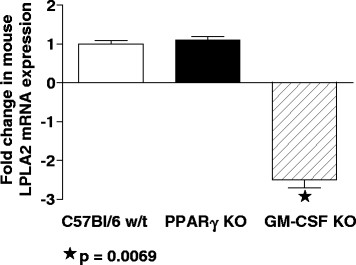
**LPLA2 gene expression is decreased in alveolar macrophages from GM-CSF KO mice as compared to wild type C57Bl6.** LPLA2 in macrophage-specific PPARγ KO mice was not different from wild type (n = 6 mice per group).

Previous studies from our laboratory and others had reported reduced expression of LPLA2 or PPARγ in GM-CSF KO mice [12,14]. Since both of these genes were deficient in pre-treatment PAP alveolar macrophages and upregulated by rituximab therapy, we sought to determine whether PPARγ might be involved in LPLA2 regulation. Therefore we investigated LPLA2 mRNA expression in BAL-derived cells from C57 wild-type, GM-CSF KO, and macrophage-specific PPARγ KO mice (Figure2). As anticipated, GM-CSF KO animals exhibited significant LPLA2 mRNA deficiency (−2.5-fold, p = 0.0069) compared to wild-type mice (Figure2). LPLA2 was not deficient, however, in macrophage-specific PPARγ KO mice which were similar to wild-type (Figure2). These data strongly suggest that LPLA2 regulation is independent of PPARγ.

### Intracellular lipid is reduced in PAP alveolar macrophages

**Figure 3:**
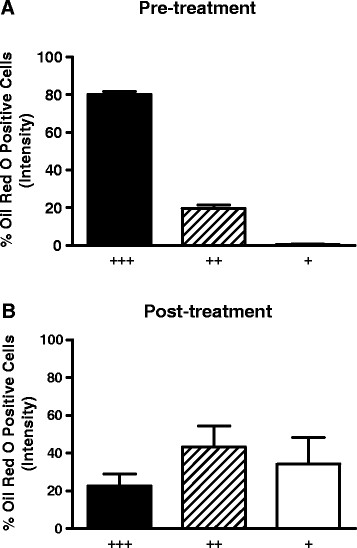
**Intracellular lipid staining is decreased post rituximab therapy in PAP alveolar macrophages.** Cytospin preparations of alveolar macrophages were stained with Oil Red O and intensity was quantified using a modified scoring previously described [21,23]; (+++ = strongly positive, ++ = positive; + = weakly positive. A minimum of 100 cells on cytospins from each of 5 paired pre and post therapy samples were scored.

Heavy (+++) intensity of Oil Red O staining characterized PAP alveolar macrophages prior to rituximab treatment (Figure3A). Post-treatment, alveolar macrophages exhibited a shift toward less Oil Red O staining, indicating a reduction of intracellular neutral lipid (p = 0.005, n = 6, Figure3B).

### Extracellular cholesterol is increased post-rituximab

**Figure 4:**
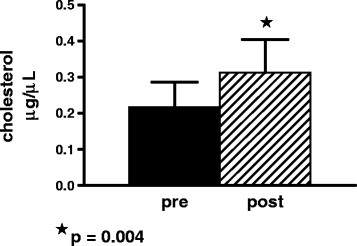
Extracellular cholesterol is increased in PAP bronchoalveolar lavage fluid post rituximab therapy (n = 8).

Because cholesterol is a component of surfactant and ABCG1 has been shown to increase cholesterol transport [27] we measured cholesterol in the BAL fluid. Rituximab increased levels of extracellular cholesterol present in PAP BAL fluid post-treatment compared to pre-treatment (p = 0.004, n = 8, Figure4).

### Surfactant protein-A (SP-A) is decreased in BAL fluid following rituximab treatment

**Figure 5:**
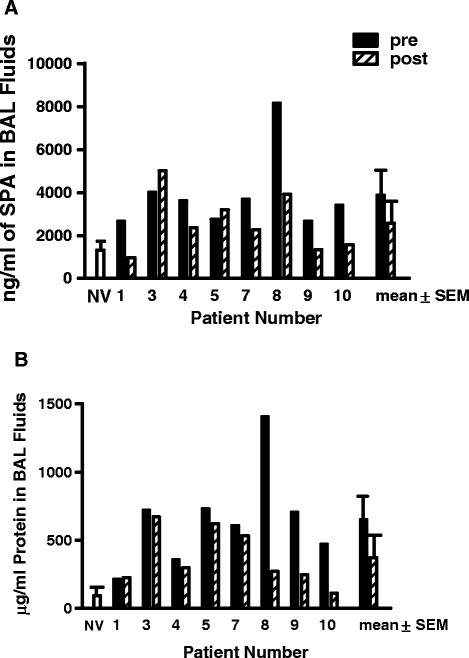
Extracellular surfactant protein A (A) and total protein (B) are decreased in PAP bronchoalveolar lavage fluid (n =8, p = 0.04 SP-A; p = 0.02 protein).

SP-A is a component is one of the four surfactant proteins and has been reported to be elevated in BAL fluid from patients with PAP [1]. Both SP-A and total protein were measured pre- and post-treatment and were found to be decreased (Figure5, n =8, p = 0.04 SP-A; p = 0.02 protein) indicating reduced extracellular accumulation of surfactant protein.

## Discussion

The findings presented here indicate that rituximab treatment can have an impact upon restoration of alveolar macrophage lipid homeostasis in PAP patients. Previous results indicated that rituximab treatment reduced anti-GM-CSF autoantibody levels in the BAL fluids of PAP patients studied here even though serum levels were not significantly affected [20]. Moreover, rituximab treatment was associated with clinical pulmonary improvement in terms of gas exchange (room air PaO_2_) and radiographic evidence of disease (by high resolution computed tomography of the chest). Data presented here confirm the positive therapeutic effects of rituximab on PAP lung with respect to enhanced alveolar macrophage functional activity and expression of lipid regulatory genes, PPARγ, ABCG1, and LPLA2. Results confirmed our initial hypothesis regarding the importance of PPARγ and ABCG1 in maintaining alveolar macrophage lipid metabolism. Compared to baseline, both of these genes were significantly upregulated by rituximab treatment. An unexpected finding was the deficiency of LPLA2 in untreated PAP patients, a situation that was significantly reversed by rituximab therapy. Our data are the first to note deficient LPLA2 in alveolar macrophages of untreated PAP patients and the upregulation of LPLA2 after rituximab therapy.

As mentioned previously in this paper, LPLA2 has also been found to be deficient in GM-CSF null mice [14]. LPLA2 activity was restored in bi-transgenic mice *in vivo* by expression of GM-CSF in type II alveolar epithelial cells under control of a surfactant protein C promoter [14]. Investigation of LPLA2 in THP-1 myeloid cells also indicated no stimulation by GM-CSF or the PPARγ ligand, Troglitazone [15].

Results of our supplemental animal studies are the first to indicate that LPLA2 is not deficient in macrophage-specific PPARγ KO mice. Thus LPLA2 deficiency in PAP does not appear to involve PPARγ although RXR is part of the PPARγ heterodimer. While these findings indicate distinct regulatory pathways for ABCG1 and LPLA2, further studies are necessary to establish the mechanisms by which GM-CSF corrects LPLA2 deficiency in PAP.

In summary, data indicate that clinical improvement post-rituximab treatment in a previously described cohort of PAP patients is associated with improved lipid homeostasis in alveolar macrophages from the same patients. Findings also indicate that reconstruction of lipid homeostasis in PAP alveolar macrophages is an intricate process requiring upregulation of the transcription factor PPARγ, lipid transporter ABCG1, and the surfactant catabolic enzyme, LPLA2. Although PPARγ dependence of ABCG1 has been established by studies in PAP and in GM-CSF KO mice [12,13,21], mechanisms regulating LPLA2 remain to be explored. Nevertheless, restoration of biologically active GM-CSF via rituximab therapy appears to be a successful means for improving lipid homeostasis in PAP alveolar macrophages.

## Abbreviations

PAP, Pulmonary Alveolar Proteinosis; GM-CSF, Granulocyte-macrophage colony stimulating factor; PPARγ, Peroxisome proliferator-activated receptor gamma; ABC, ATP binding cassette; ABCG1, ABCA1, Lipid transporter; LPLA2, Lysosomal phospholipase A2.

## Competing interests

Genentech funded the clinical trial.

## Authors’ contributions

AM contributed to acquisition of the data, analysis and interpretation of data, drafting of the manuscript and final approval of the version to be published; MSK contributed to the conception and design, acquisition of the data, analysis and interpretation of data and final approval of the version to be published, IM contributed to acquisition of the data and final approval of the version to be published; BPB contributed to the design, analysis and interpretation of data, drafting of the manuscript and final approval of the version to be published; IH contributed to the acquisition and interpretation of data and final approval of the version to be published; RK contributed to the acquisition of data and final approval of the version to be published; MJT contributed to the conception and design, acquisition of data, analysis and interpretation of data, drafting of the manuscript and final approval of the version to be published. All authors read and approved the final manuscript.

## Funding sources

Supported by Genentech and the National Institute of Health in part by grant RO1-AI064153.
